# Road traffic delays in commuting workplace and musculoskeletal health among sedentary workers: A cross‐sectional study in Dhaka city

**DOI:** 10.1002/1348-9585.12289

**Published:** 2021-11-09

**Authors:** Mohammad Ali, Gias U. Ahsan, Zakir Uddin, Ahmed Hossain

**Affiliations:** ^1^ Department of Physiotherapy and Rehabilitation Uttara Adhunik Medical College and Hospital Dhaka Bangladesh; ^2^ Centre for Higher Studies and Research Bangladesh University of Professionals Dhaka Bangladesh; ^3^ Department of Public Health North South University Dhaka Bangladesh; ^4^ School of Physical and Occupational Therapy McGill University Montreal Quebec Canada; ^5^ School of Rehabilitation Sciences McMaster University Hamilton Ontario Canada; ^6^ Global Health Institute North South University Dhaka Bangladesh

**Keywords:** Bangladesh, commuting, low back pain, musculoskeletal health, road traffic delays, sedentary workers, traffic congestion

## Abstract

**Objectives:**

Despite previous research aimed at identifying factors linked to musculoskeletal health issues, there was no evidence about the relationship between road traffic delays (RTDs) and musculoskeletal health in sedentary employees. As a result, the aim of our research was to understand such a correlation among bank employees in Dhaka, Bangladesh.

**Methods:**

A cross‐sectional analysis was conducted with bank employees who worked in sedentary settings. The Eriksen subjective health complaints scale was used to measure the eight items of musculoskeletal health complaints (MHCs), and RTDs were measured using principal component analysis using variables commute time, distance, and traffic congestion experience to work. The association between RTDs and MHCs was identified using a multilevel model after adjusting potential confounders.

**Results:**

A total of 628 employees (mean[SD] age, 36.1[7.0] years; 254[40.5%] women) participated in the study. Among the employees, the one‐month prevalence of MHCs was 57.2%. The highest prevalence of MHCs was low‐back pain (36.6%), followed by neck pain (22.9%) and upper‐back pain (21.2%). Also, 136(21.7%) employees reported long‐RTDs in commuting workplace and 81% of them had MHCs. The multilevel analysis identified that long‐RTDs had a significant relationship with MHCs (adjusted odds ratio, AOR = 10.20, 95%CI = 5.41–16.91). Private transportation commuters reported 70% reduced odds of MHCs (AOR = 0.30, 95%CI = 0.15–0.59) and walking or bicycling commuters had 84% fewer MHCs (AOR = 0.16, 95%CI = 0.10–0.28) compared to public bus commuters.

**Conclusions:**

Sedentary employees with long‐RTDs reported increased MHCs, emphasizing the importance of including musculoskeletal exercise in office facilities. Findings of this study also highlight the need for a sound public transportation system in Dhaka city.

## 1 INTRODUCTION

Increasing traffic congestion in large and growing cities around the world is an inescapable situation. Owing to peak‐hour traffic congestion, office commuters suffer the brunt of lengthy delays. Owing to road traffic delays (RTDs), long wait times can be left on the streets, which has a negative effect on the economy. The delays cause office transitions over an extended period to adopt a long duration and the same posture in a vehicle, which can trigger musculoskeletal problems.^1^, ^2^, ^3^

Most commuters globally suffer health problems due to RTDs. A longitudinal study in the United Kingdom demonstrated that long commuting time is related to low subjective health condition, low health satisfaction, poor sleep quality, and high body mass index.^4^ Another study found long commuting time related to worse mental health.^5^ A study conducted in Norway found a high prevalence of subjective health complaints among long commuting railway workers.^6^ Furthermore, long‐distance commuting affects physical activity, cardiorespiratory health, adiposity, and metabolic risk indicators.^7^

Long‐distance commuters are often subjected to concentrations of air pollution and noises for a long time, with commuters stuck in congested traffic bearing the brunt of the burden. Prolonged and regular exposure to noise and air pollution can produce anxiety, depression, sleep disturbance, anger, and displeasure.^8^, ^9^, ^10^ Commuters feel stressed and become anxious during the long commuting time.^11^, ^12^ This type of anxiety and depression due to long commuting time could exacerbate musculoskeletal problems.^13^, ^14^ Despite a modest rise in traffic volume, RTD exposures such as air pollution or noise pose a significant health danger, and it has a significant effect on public health.^15^, ^16^, ^17^

Dhaka, the capital city of Bangladesh, is one of the most traffic‐congested cities in the world.^18^ Office commuters in Dhaka city experience an inevitable overload on existing roads and transit systems every day.^18^ The first thing the commuters think of when it comes to congested roadways is the delay. The current road system cannot handle peak‐hour loads without forcing many people to wait in line for limited road space, and it causes road traffic delays to workplace.^18^

Sedentary work is any form of work that involves much of the time spent sitting with little or intermittent walking or standing. The work of most bank workers is sedentary in nature. When they carry out their jobs, these individuals spend nearly all their working hours sitting. The majority of studies have shown that sedentary habits are a risk factor for low back pain (LBP).^19^, ^20^ A considerable number of bank workers have long commutes to work due to traffic congestion, and they spend a significant amount of time sitting in transportation. Sitting for long periods of time can affect the spine and cause other health problems.

The MHCs may be due to RTDs to workplace that have never been studied among the full‐time sedentary workers in Dhaka City. Thus, the study has two objectives: (1) to estimate the prevalence of MHCs among Dhaka city bank employees, and (2) to identify the relationship between RTDs and MHCs.

## 2 MATERIALS AND METHODS

### 2.1 Study site: Dhaka city

Dhaka is one of the world's top twenty most populated cities, with a population of about 21 million that is rapidly increasing every year.^21^ People are likely to continue to migrate to Dhaka city for a variety of reasons, including convenience, better employment, easy access to social services, and higher wages. Dhaka is a megacity where there are lots of commuters on the road, and most of the commuters experience heavy traffic congestion every day. Overcrowded public transports are a common sight in Dhaka.^22^ There are more than 500,000 rickshaws, along with approximately 1.1 million registered motor vehicles operating every day in the city.^23^

### 2.2 Study design and settings

This study was carried out from January 2019 to May 2019 among Bank employees in Dhaka city of Bangladesh. Bangladesh currently has 61 scheduled banks, with 9 being state‐owned commercial banks and the other 53 being private commercial banks. In Dhaka, every bank has many branches. We collected data from 32 banks by distributing questionnaire to 82 branches. To define a study participant from a bank, we used the following inclusion and exclusion criteria.

### 2.3 Inclusion criteria

- Full‐time bank employees who were working in banks for at least one year,
- Age: 18–59 years old,
- Bank employees who worked in a sedentary setting that they spent 80% of their time sitting,
- Those who completed the questionnaire and signed up the consent form to participate in the study.

### 2.4 Exclusion criteria

1. Pregnant workers or female workers who had a baby less than six months of age,
2. Workers with any history of spinal surgery or surgery in the pelvic region,
3. Workers with any experience of back injuries or any other spine injury due to accident (e.g., road accident).
4. Any history of joint or bone disorder or prolapse lumbar intervertebral disc (PLID).
5. Any history of chronic inflammatory pain (e.g., rheumatoid arthritis, ankylosing spondylitis).

### 2.5 Sampling technique and sample size

A convenience sampling technique was used to select the banks and employees following the STROBE guideline. The minimum necessary sample size for the study was calculated based on a 95% confidence interval (CI) and assuming the prevalence of LBP among full‐time employees as 35%. We calculated the minimum required sample as 546 by considering a 4% marginal error.

We distributed the questionnaire to the bank employees from 83 branches of 32 banks in Dhaka city. We described the questionnaire in both English and Bengali languages for a better understanding and factual answering of the participants. The 923 bank employees who met the eligibility criteria were given a paper‐based questionnaire, and 652 of them returned the questionnaire within 7 days after distribution. However, 628 participants completed the questionnaires, and we entered the data with an undisclosed identification number for each participant in a personal computer for analysis.

## 3 STUDY VARIABLES

### 3.1 Measurement of musculoskeletal health complaints

The questions on musculoskeletal symptoms were based on the subscale of subjective health complaints by Eriksen et al. that measure health complaints experienced during last 30 days.^24^, ^25^ In this subscale of 8 items (shoulder pain, neck pain, lower back pain, upper back pain, arm pain, headache, leg pain during physical activity, and migraine) are included for which the severity of each complaint is scored on a 4‐point scale ranging from 0 (no complaint) to 3 (severe complaints). Thus, employees were asked to rate the occurrence of pain or discomfort using the 8 items with four answering categories (“no complaint,” “only once/a little,” “of short duration/ some,” “frequently/ serious”). Employees who answered, “no complaint,” “only once/a little” on all questions were classified as having no musculoskeletal health complaints. Those who answered “of short duration/ some” or “frequently/ serious” for one or more locations were classified as having musculoskeletal health complaints overall.

### 3.2 Independent variables

Data on socio‐demographic factors—age, gender, BMI (weight‐to‐height ratio), and marital status were collected using a semi‐structured questionnaire. Overweight was defined as a BMI more than or equal to 25 but less than 30, and obesity was defined as a BMI equal to or greater than 30. Behavioral factors including sleep arrangements (firm or foam mattresses), smoking habits (current, previous, or never), and physical activities of the respondents were collected. The response of sleep arrangement by a firm or foam mattress was subjective about the feel of rigidness about the mattress. Physical activities were calculated based on the metabolic equivalents (MET minutes/week) scale. In this study, the levels of physical activity of the respondents were measured by asking about their weekly activities during work and leisure time, activities related to transport, and time spent in a sedentary position. MET‐minute was calculated according to the STEPS protocol, and physical activity was categorized into moderate to vigorous, light, and sedentary activity.^26^ We also collected data on occupational factors, including the length of employment and average daily working hours. The crowding was calculated by dividing the number of family members in the house by the number of bedrooms. We categorized the in‐housing crowding in three groups: <=1.5, 1.5–2.0, and >2.0. Data on common chronic illness (diabetes and hypertension) from the employees were also collected.

### 3.3 Road traffic delays index

Road traffic delays (RTDs) was defined as the unnecessary travel time.^22^ Therefore, the estimation of the delay equations requires several considerations. We took three factors to calculate the RTDs: (1) commute time to workplace (minutes), (2) commuting distance (km) to workplace, and (3) overall subjective traffic congestion experience (yes / no). The coding of the 3 factors as categorical variables is given in the Supplement S1. We generated the RTDs index using a principal component analysis and place individuals on a continuous scale‐based on the scores of the first principal component. First principal component explains the largest proportion of the total variance and it was used as the RTDs index. The scale was then ranked, after which it was subdivided into 5 equal stratums called RTD quintiles. The lowest two‐quintile (quintiles 1 and 2) represent the no delay or minimum delay, the participants belong to upper two quintiles (quintiles 4 and 5) were in the category of long‐RTDs, and the rest (quintile 3) considered to the category of moderate‐RTDs.

### 3.4 Statistical analysis

The questionnaire and data are available at https://osf.io/6bq7z/. Data were analyzed using R 3.6.2. Descriptive statistics were calculated and presented as frequencies and percentages for all the group variables. We performed a directed acyclic graph (DAG) for the adjustment of confounders in the associations between RTDs to workplace and MHCs. This graphical approach illustrates hypothesized causal relationships and deduces the correlations that these causal relationships suggest. We consider MHCs as the outcome variable, and the main exposure is RTDs to the workplace. The plot indicates that age, gender, BMI, and commuting transport to the workplace are the minimum adjustment sets for estimating the direct effect of exposure to MHCs. This figure was constructed through DAGITTY (http://www.dagitty.net/dags.html#) and is given in the Supplement S2. We fitted a multilevel logistic model with the presence of MHCs. To run the multilevel logistic model, we chose a random intercepts model, with fixed slopes using the “glmer()” function from the “lme4” package of R. Here we specified the intercept varies by banks. The results are reported by the adjusted odds ratios (ORs), and corresponding p‐values are also presented in a table. *p*‐values less than .05 were considered statistically significant. The percentage of missing data in the dataset ranged from 0.1% for the age group to 0.3% for the RTDs. In multivariable analysis, we did not consider the two participants that had these missing data points.

## 4 RESULTS

### 4.1 Characteristics of the participants

The mean ±SD age of the 628 participants was 36.1 ± 7 years. Table 1 shows the descriptive statistics of the socio‐demographic factors and behavioral factors by MHCs. We had 499 (79.3%) of the respondents were young adults (20–40 years of age), and 373 (59.5%) of the respondents were male. Age appears to be significant at the 5% significance level in the unadjusted analysis. Despite the fact that gender did not exhibit significance in the unadjusted analysis, female employees complained (61%) more about musculoskeletal problems than male employees (55%) in the study. It appears that 51.8% of adults between the ages of 20 and 30 reported at least one MHCs, while 68% of adults aged 40 and up reported at least one MHCs. Among the participants, 516 (82.3%) were married, and 307 (59.5%) of the married employees reported MHCs. Marital status is significant at a 5% level in the unadjusted analysis. Among the respondents, 320 (51%) were in normal weight, 269 (42.9%) were overweight, and 36 (6.1%) were obese. Obese employees reported (71%) more complaints on musculoskeletal health compared to the overweight (58.4%) or healthy‐weight (54.7%) participants.

**TABLE 1:** Univariate analysis: Socio‐demographic and behavioral factors, and MHCs

Factor	Categories	Musculoskeletal health complaints (MHCs)		Total (%) within categories	*p*‐value*
		Yes (Row %)	No (Row %)		
Age group	20–30	72 (51.8)	67 (49.2)	139 (22.1%)	.**044**
	31–40	200 (55.6)	160 (44.4)	360 (57.2%)	
	41–50	68 (68)	32 (32)	100 (15.9%)	
	50+	20 (71.4)	8 (28.6)	28 (4.5%)	
Gender	Male	205 (54.9)	168 (45.1)	373 (59.5%)	.185
	Female	154 (60.6)	100 (39.4)	254 (40.5%)	
BMI	Normal (18.5–24.99)	175 (54.7)	145 (44.3)	320 (51.0%)	.138
	Overweight (25–29.99)	157 (58.4)	112 (41.2)	269 (42.9%)	
	Obese (>=30)	27 (71)	11 (29)	38 (6.1%)	
Marital Status	Married	307 (59.5)	209 (40.5)	516 (82.3%)	.**019**
	Unmarried	52 (46.8)	59 (43.2)	111 (17.7%)	
In‐house Crowding	≤ 1.5	185 (55.6)	148 (44.4)	333 (53.1%)	.654
	1.5–2	117 (59.4)	80 (40.6)	197 (31.4%)	
	2+	57 (58.8)	40 (41.2)	97 (15.5%)	
Sleeping mattress	Firm bed	302 (57.1)	227 (42.9)	529 (84.4%)	.931
	Foam bed	57 (58.2)	41 (41.8)	98 (15.6%)	
Smoking habit	No	301 (58.3)	215 (41.7)	516 (82.3%)	.285
	Yes	58 (52.3)	53 (47.7)	111 (17.7%)	
Physical Activity	Sedentary	115 (59.9)	77 (40.1)	192 (30.6%)	.674
	Light	213 (56.1)	167 (43.9)	380 (60.6%)	
	Moderate to vigorous	31 (56.4)	24 (43.6)	55 (8.8%)	
Diabetes/ Hypertension	No	318 (56.9)	241 (43.1)	559 (89.1%)	.685
	Yes	41 (60.3)	27 (39.7)	68 (10.9%)	

* *p*‐value is calculated from chi‐square test.

Bold faces represent significant at 5% significance level.

### 4.2 Prevalence of MHCs

**FIGURE 1:**
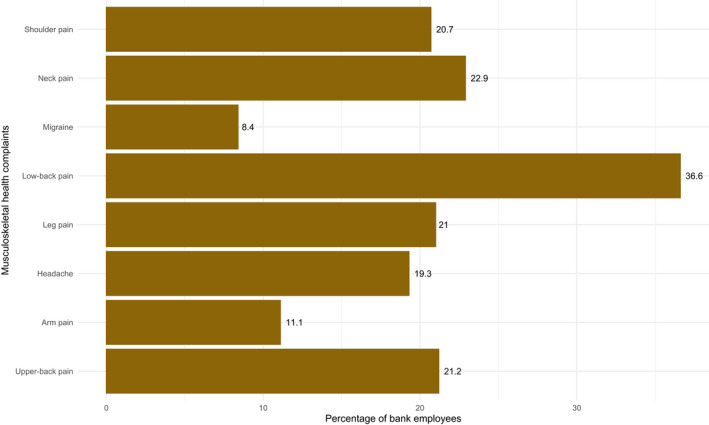
Prevalence of musculoskeletal health complaints among bank employees

We showed the one‐month prevalence of MHCs among the 628 bank employees in Figure 1. Overall, 359 (57.2%) of the employees reported experience of at least one musculoskeletal pain during the last one month. Among the eight MHCs, the prevalence of low back pain (LBP) was the highest (36.6%), followed by neck pain (22.9%) and upper back pain (21.2%). Moreover, 19.3% of the employees reported headache, and 8.4% of the bank employees reported migraine during the last one month.

### 4.3 Descriptive analysis: occupational factors and RTDs

The results of MHCs by occupational factors and RTDs are given in Table 2. The majority of workers tend to have commuted through public transportation. The results show that 226 (36%) of the 627 workers used public transportation to get to work, and 80.5% of these 226 workers reported musculoskeletal pains. In addition, 13.4% of workers stated that they walked or rode their bikes to work, and 33.3% of them said that they had musculoskeletal problems. Another 193 (30.8%) of bank employees said that they took a rickshaw to work, with 43.8% of them complaining of musculoskeletal problem. The variable commuting transport is significant at the 5% significance level in the unadjusted analysis. Employees’ long employment history was found as a significant variable with MHCs. Furthermore, 258 (41.1%) of the 627 employees said that they worked long hours (>9 h) at workplace, with 159 (61.6%) of them reporting at least one musculoskeletal issue. There were 187 employees (29.8%) who said that they had a long commute to work (more than one hour), and 148 (79.1%) of them reported musculoskeletal problems. Furthermore, 210 (33.5%) of participants said that they commuted more than 9 km to work each day, and 159 (75.7%) of them reported musculoskeletal complications. Furthermore, 344 (54.9%) of the participants said that they had experienced frequent traffic congestion when commuting to work, and 281 (81.7%) of them reported musculoskeletal problems. The RTDs index was estimated using travel time, commuting distance, and traffic congestion experience. The table shows 136 (21.7%) of the employees reported long RTDs to work, and 111 (81.6%) of these 136 workers reported musculoskeletal problems. However, 109 workers (17.4%) did not mention RTDs, and 26 (23.9%) of them had musculoskeletal complications.

**TABLE 2:** Univariate analysis: occupational factors and RTDs factors with MHCs

Factor	Categories	Musculoskeletal health complaints		Total (%) within categories	*p*‐value*
		Yes (Row %)	No (Row %)		
Job duration (years)	≤5	109 (50.5)	107 (49.5)	216 (34.4%)	.**006**
	6–10	116 (56)	91 (44)	207 (33.0%)	
	10+	134 (65.7)	70 (34.5)	204 (32.6%)	
Working hours/day	Extended (>9)	159 (61.6)	99 (38.4)	258 (41.1%)	.077
	Regular (8–9)	200 (54.2)	169 (35.8)	369 (59.9%)	
Average commute time to office (minutes)	≤ 15	40 (33.6)	79 (36.4)	119 (19.0%)	**<.001**
	16–30	55 (33.1)	111 (36.9)	166 (26.5%)	
	31–60	116 (74.8)	39 (25.2)	155 (24.7%)	
	>60	148 (79.1)	39 (20.9)	187 (29.8%)	
Distance to office (Kilometer)	≤2	55 (32.2)	116 (67.8)	171 (27.3%)	**<.001**
	3–5	73 (51.0)	70 (49)	143 (22.8%)	
	6–8	72 (69.9)	31 (30.1)	103 (16.4%)	
	≥9	159 (75.7)	51 (24.3)	210 (33.5%)	
Commuting transport	Public bus	182 (80.5)	44 (19.5)	226 (36.0%)	**<.001**
	Private transport (Car)	35 (58.3)	25 (41.7)	60 (9.5%)	
	Rickshaw	84 (43.5)	109 (56.5)	193 (30.8%)	
	Walk/bicycle	28 (33.3)	56 (56.7)	84 (13.4%)	
	Others (train, motor cycle, autorickshaw, etc.)	30 (46.9)	34 (53.1)	64 (10.2%)	
Experience of regular traffic congestions	No	78 (27.6)	205 (72.4)	283 (45.1%)	**<.001**
	Yes	281 (81.7)	63 (18.3	344 (54.9%)	
Road traffic delay (RTD) index	No or Low delay	26 (23.9%)	83 (76.1)	109 (17.4%)	**<.001**
	Moderate delay	222 (58.1%)	160 (41.9%)	382 (60.9%)	
	Long delay	111 (81.6%)	25 (18.4%)	136 (21.7%)	

* *p*‐value is calculated from chi‐square test.

Bold faces represent significant at 5% significance level.

### 4.4 Descriptive analysis: association between age, gender, BMI, RTDs, and commuting mode

**FIGURE 2:**
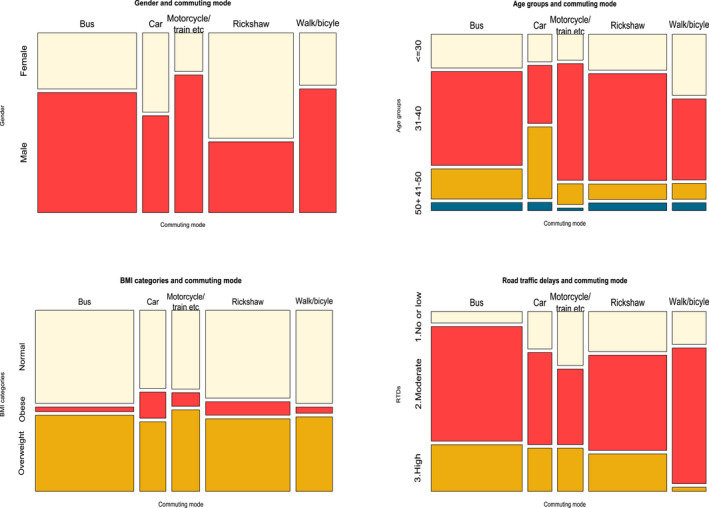
Mosaik plot to understand association between age, gender, BMI categories, RTDs, and commuting mode

To better understand the association between age, gender, BMIs, RTDs, and commuting mode, see Figure 2. The majority of employees commuted to work by bus or rickshaw. The majority of bus commuters appeared to be male, and male employees also walked or rode bicycles for commuting more than female employees. Employees aged 40 and up were the majority of those who used the private transports. Obese employees mostly avoided taking the bus to work. Those who took the bus also complained about traffic congestion, long commute, and long‐time commute.

### 4.5 Multilevel logistic regression model

Table 3 shows the results of the adjusted odds ratio from a mixed logistic model with a random intercept. We allow the intercept to vary randomly by each bank. The mixed logistic regression model was used to identify the effect of road traffic delays on MHCs after adjusting age, gender, BMI, and commuting transport variables. To establish the minimum number of adjusted variables, the DAG plot was employed (see Supplement S2). The results indicate that the employees with long‐RTDs had higher odds of MHCs compared to the no or low‐RTDs (adjusted odds ratio, AOR = 10.15, 95% CI = 5.37–19.86). Moreover, the odds of bank employees with moderate‐RTDs had 3.43 times more MHCs compared to the employees with no or low‐RTDs (AOR = 3.43, 95% CI = 2.06, 5.85). The significance of RTDs indicates that the long‐time commuting or long‐distance commuting had a significant effect on the high prevalence of MHCs. In the analysis, employees using a private transport as the commuting mode can reduce 70% of the odds of developing MHCs compared to the employees who used a public transport (AOR = 0.30, 95% CI = 0.15– 0.59). Walking or using bi‐cycle to commute workplace can reduce 84% of odds of developing musculoskeletal health problems compared to using a public transport for commuting (AOR = 0.16, 95% CI = 0.10–0.28).

**TABLE 3:** Adjusted analysis by a random intercept logistic regression model

Variables	Categories	Unadjusted OR	Adjusted OR
Age groups	<=30 Years	Reference	
	31–40	1.16 (0.79–1.72)	1.21 (0.76– 1.91)
	41–50	**1.97 (1.15–2.38)**	1.61 (0.86–3.04)
	50+	**2.19 (1.21–4.64)**	**2.03 (1.01–5.18)**
Gender	Male	Reference	
	Female	**1.26 (0.91–1.74)**	**1.69 (1.14–2.53)**
BMI category	Normal	Reference	
	Overweight	1.16 (0.84–1.61)	1.21 (0.83–1.76)
	Obese	2.03 (0.98–4.24)	**2.35 (1.07–5.49)**
Road traffic delays	No or minimum delay	Reference	
	Moderate delay	**4.29 (2.73–7.19)**	**3.41 (2.06–5.81)**
	Long delay	**14.17 (7.64–19.30)**	**10.21 (5.40–16.81)**
Commuting transport	Public bus	Reference	
	Private transport (Car)	**0.34 (0.18–0.62)**	**0.33 (0.17– 0.64)**
	Motorcycle or Train	**0.21 (0.12–0.39)**	**0.26 (0.13–0.49)**
	Rickshaw	**0.19 (0.12–0.29)**	**0.17 (0.10–0.27)**
	Walk/ Bicycle	**0.12 (0.07–0.21)**	**0.16 (0.09–0.28)**

Note

Bold faces present as significant variable at 5% significance level.

[Table 3: Adjusted odds ratios from mixed logistic regression model after adjusting confounders.]

### 4.6 Sensitivity analysis

We also employed three binary logistic regressions for examining (i) the relationship between MHCs and commuting time to workplace, (ii) the relationship between MHCs and distance to commute workplace, and (iii) the relationship between MHCs and experience of traffic congestion to commuting workplace. The results of these three models are given in the Supplement S3–S5, respectively. The models explain how specific RTD factors exhibited significance to MHCs at 5% significance level. We also obtained variance inflation factors (VIF) in the logistic regression models to evaluate potential multicollinearity. The results indicate that commute time more than 30 min and commuting distance more than 2 kilometers play a significant role in developing musculoskeletal health problems. For example, employees with commute times of more than one hour were 7.32 (aOR = 7.32, 95 percent CI = 3.57–15.03) times more likely to had musculoskeletal health problems than employees with commute times of 15 min or less. Also, the employees who reported traffic congestions in commuting workplace complained significantly more musculoskeletal health problems than who did not experience traffic congestions (aOR = 13.0, 95% CI = 7.7–22.1).

## 5 DISCUSSION

The study reveals that the one‐month prevalence of MHCs among full‐time bank employees was 57.2%. According to a study conducted in China, 68.3 percent of health‐care professionals suffer from musculoskeletal disorders (MSDs).^27^ Another study in Saudi Arabia indicated that 56% of the adult population were in chronic musculoskeletal pain.^28^ Moreover, the one‐year prevalence of MSDs among Iranian office workers was 52.8%^29^ and among Thai office workers was 63%.^30^ The prevalence of MSD was 70.5% among taxi drivers in Ghana and 80% of bank workers in Kuwait, suggesting a high prevalence of MSD among these two occupational groups.^31^, ^32^

In our study, the one‐month prevalence of low back pain (LBP) was 36.6%, and neck pain was 22.9%. A study in Iran showed that one‐year prevalence rate of LBP was 57.1% among office workers.^29^ However, the prevalence of neck pain was leading MSD (60.2%) among Iranian office workers, which is much higher than our findings.^29^ A meta‐analysis showed that the prevalence of musculoskeletal problems (MSP) among bus drivers varied from 43.1% to 93%.^33^ Low back pain was the most commonly reported body area for MSP (53%), and the neck, shoulder, and upper back were the other prominent regions with high prevalence.^33^ However, three studies conducted in Bangladesh found that the prevalence of LBP was 72.9% and 36.6% among nurses and bank employees, respectively.^34^, ^35^ Many of these workers who worked in sedentary environments had a high prevalence of musculoskeletal disorders.

We found a strong positive association between road traffic delays to workplace and MHCs. Studies in Norway, Ghana, and Malaysia reported that long‐commute to workplace and traffic congestions are associated with musculoskeletal problems.^6^, ^36^, ^37^ There is a clear positive correlation between long‐commute time to the workplace and MHCs.^36^, ^37^ Long‐distance commute to the office is also positively linked to MHCs and is also listed in a few studies.^6^, ^31^, ^36^ Moreover, we found that employees who walked or used private transport, rickshaw, or cycle to work were found to be more satisfied with their musculoskeletal health than those who used public buses. A study reported commuting by a transport has an impact on overall well‐being and suggested cycling and walking for better well‐being.^37^ Many commuters in Dhaka city are used to take their daily rides on public buses. The public transports like buses from government fleet are the most fitting for Dhaka, where millions of passengers have to ride every day in various directions. Both the low‐floor regular and double‐decker buses can ensure a decent travel which is entirely absent in many public buses running on the roads.

The associated factors of MHCs among full‐time bank employees are also noted in our study. The findings of this study showed that among office workers, older age, female employees, and obesity were positively correlated with MHCs. We found that the MHCs were more common among female employees than among male employees. A research in Germany reported the point prevalence of back pain for women was 40% and for men was 32%.^38^ The association between gender and MHCs may be too simplistic and further evaluation may be required. This problem may be influenced by assumed confounding variables such as pregnancy, a primary care role in household or domestic responsibilities. We noticed that physical activity was not a priority for bank employees, with only 8.8% engaged in moderate to severe physical activity, preventing us from determining the influence of physical activity on musculoskeletal problems. However, this variable was found significant in a meta‐analysis suggesting that exercise alone may reduce the risk of LBP.^39^ Also, the lack of importance of physical activity in our study could be due to the fact that we used the employees’ current physical activity rather than their history of physical activity. For most of office commuters, cutting the commute may not be an option, whether using a bus or train ride to work. The first and most important thing could be physical exercise and opening up options for occupational exercise in workplace. Another reasonable alternative is to get off the bus one or two stops earlier and walk to the office.

## 6 LIMITATIONS AND STRENGTH

One of the limitations of this research is its cross‐sectional research design, which cannot establish the causative relationship between RTDs to workplace and MHCs. Other limitations of the study include a lack of information about workload details, work stress, and seating structure ergonomics in workplace. Such limitation in analysis was reduced by using a homogeneous working community of people who work in sedentary environments. The factors RTDs and commuting transportations and the outcome were measured subjectively in this study. We were also unable to provide comprehensive details on chronic pain or severity of pain, or whether or not the workers were visiting a doctor because of the pain. The importance of socioeconomic status in identifying the association between RTDs and MHCs cannot be disregarded, which is currently not been adjusted in the article. However, by using a homogeneous sample of bank employees in terms of education and income, we were able to reduce the bias caused by SES variability. The research's strength is that it used a homogeneous population with similar types of employment, a common sitting structure, and a similar work climate. Our study is the first to show a correlation between RTDs to the workplace and MHCs in sedentary workers of a populous city like Dhaka. Any future research should include other occupational groups in order to gain a broader image across the multi‐professional sedentary workers.

## 7 CONCLUSIONS

The study found a high burden of musculoskeletal complaints among full‐time bank employees in Dhaka city, and low back pain was the most prevalent complaint. Road traffic delays to workplace in the Dhaka city is common and have adverse effects on musculoskeletal problems. The factors of RTDs such as long‐distance commuting, log‐time commuting and traffic congestions should be included in a discussion between government and health policy maker to minimize musculoskeletal health issues for workers. Current practice of public bus commutes is more prone to endure musculoskeletal pains and so public transportation should be expanded in the city to accommodate more passengers in a comfortable manner. Testing the feasibility by replacing private transports on a few city routes could bring a favorable result on health issues of commuters. The high prevalence of MHCs also emphasizes the value of including musculoskeletal fitness in office training to increase awareness and prevent health issues.

## DISCLOSURE

Ethical approval: The ethical committee of the Bangladesh University of Professionals (2019/273) and IRB of North South University (NSU‐IRB‐2019–54) approved the study. The objectives of the study, along with its procedure, were explained to the respondent, and signed informed consent was taken from each respondent. Registry and the Registration No. of the study/Trial: N/A. Animal Studies: N/A. Consent to Publish: Not applicable.

## CONFLICT OF INTEREST

The authors declare that they have no conflict of interest.

## AUTHOR CONTRIBUTIONS

MA and AH participated in the study conception, design, and coordination of the manuscript. GUA and ZU reviewed the manuscript and helped to draft the manuscript. AH also performed the statistical analysis and helped to draft the manuscript. All authors approved the final manuscript.

## Supporting information

Supplementary Material

## Data Availability

The data that support the findings of this study are openly available in Open Science Forum: https://osf.io/6bq7z.
