# Supplementary material for: Road traffic delays in commuting workplace and musculoskeletal health among sedentary workers: A cross‐sectional study in Dhaka city
Source: J Occup Health. 2021 Nov 9;63(1):e12289. doi: 10.1002/1348-9585.12289 (PMC8577272; doi:10.1002/1348-9585.12289)

Table of Contents

[S1: Coding of factors for road traffic delays. 1](#_Toc78926088)

[S2: DAGITTY plot 2](#_Toc78926089)

[S3: Multivariable logistic regression model: Adjustment of confounders including commuting time to workplace 3](#_Toc78926090)

[S4: Multivariable logistic regression model: Adjustment of confounders including commuting distance to workplace 4](#_Toc78926091)

[S5: Multivariable logistic regression model: Adjustment of confounders including traffic congestion experience to workplace 5](#_Toc78926092)

# S1: Coding of factors for road traffic delays.

| **Name of the Variable** | **RTDs** | Values |
| --- | --- | --- |
| Commute time to workplace | <15 minutes | 1 |
|  | 16-30 minutes | 2 |
|  | 31-60 minutes | 3 |
|  | >60 minutes | 4 |
|  |  |  |
| Commuting distance (km) to workplace | <=2 km | 1 |
|  | 2.0-5 km | 2 |
|  | 5.0-8 km | 3 |
|  | >8 km | 4 |
|  |  |  |
| Overall subjective traffic congestion experience | Yes | 1 |
|  | No | 0 |

# S2: DAGITTY plot

**Figure.** DAG demonstrating causal relationships and potential biasing pathways affecting the association between road traffic delays and musculoskeletal health complaints (produced using DAGitty V.2.3 software). In this conceptual diagram, each circle represents an individual exposure (‘node’) of theoretical relevance to this hypothesis; each node is interconnected by directional arrows (‘edges’) that represent theoretical associations based on the researchers’ assessment of a priori literature and determination of biological plausibility. RTDs and commuting transport were the exposure of interest (green node with black border), with MHCs (blue node with black border) as the outcome of interest. In this instance, all the other exposures (‘nodes’) are theoretically causally associated with (i.e., ancestors of) both the exposure and the outcome. To adjust for confounding in the association of interest, it is necessary to close all ‘backdoor pathways’ between the exposure and outcome (i.e., any pathway (consisting of a series of one or more edges and nodes) that provides an alternate route between the exposure and outcome); this is accomplished by adjusting for at least one node on that path. The minimally sufficient adjustment set is the combination of the fewest nodes that, being ancestors of both the exposure and outcome, if selected, effectively block all backdoor pathways between the exposure and the outcome (white nodes with black borders). These ‘adjusted variables’ are then introduced into the multivariate modelling as potential confounders.

# S3: Multivariable logistic regression model: Adjustment of confounders including commuting time to workplace


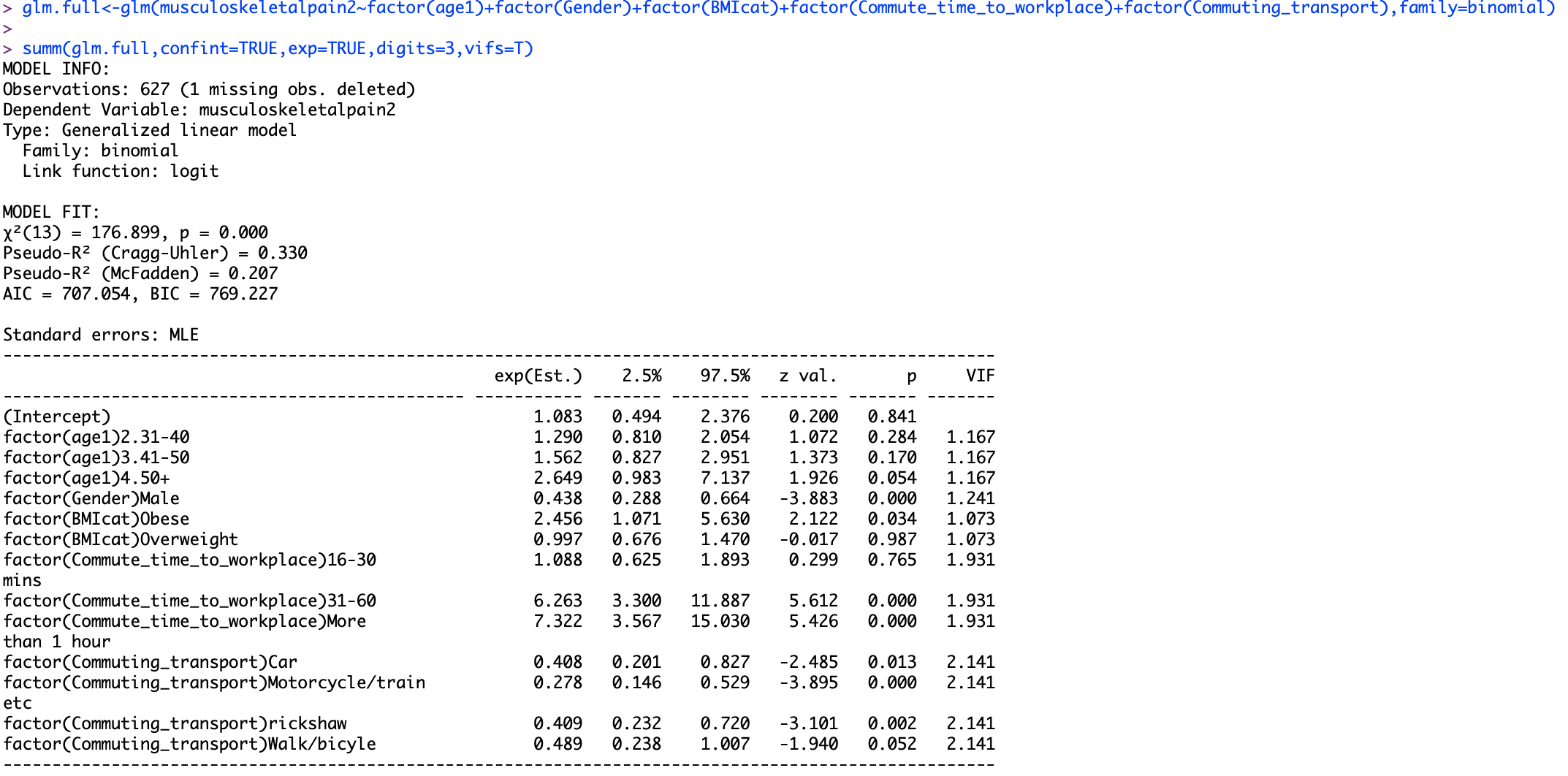


# S4: Multivariable logistic regression model: Adjustment of confounders including commuting distance to workplace


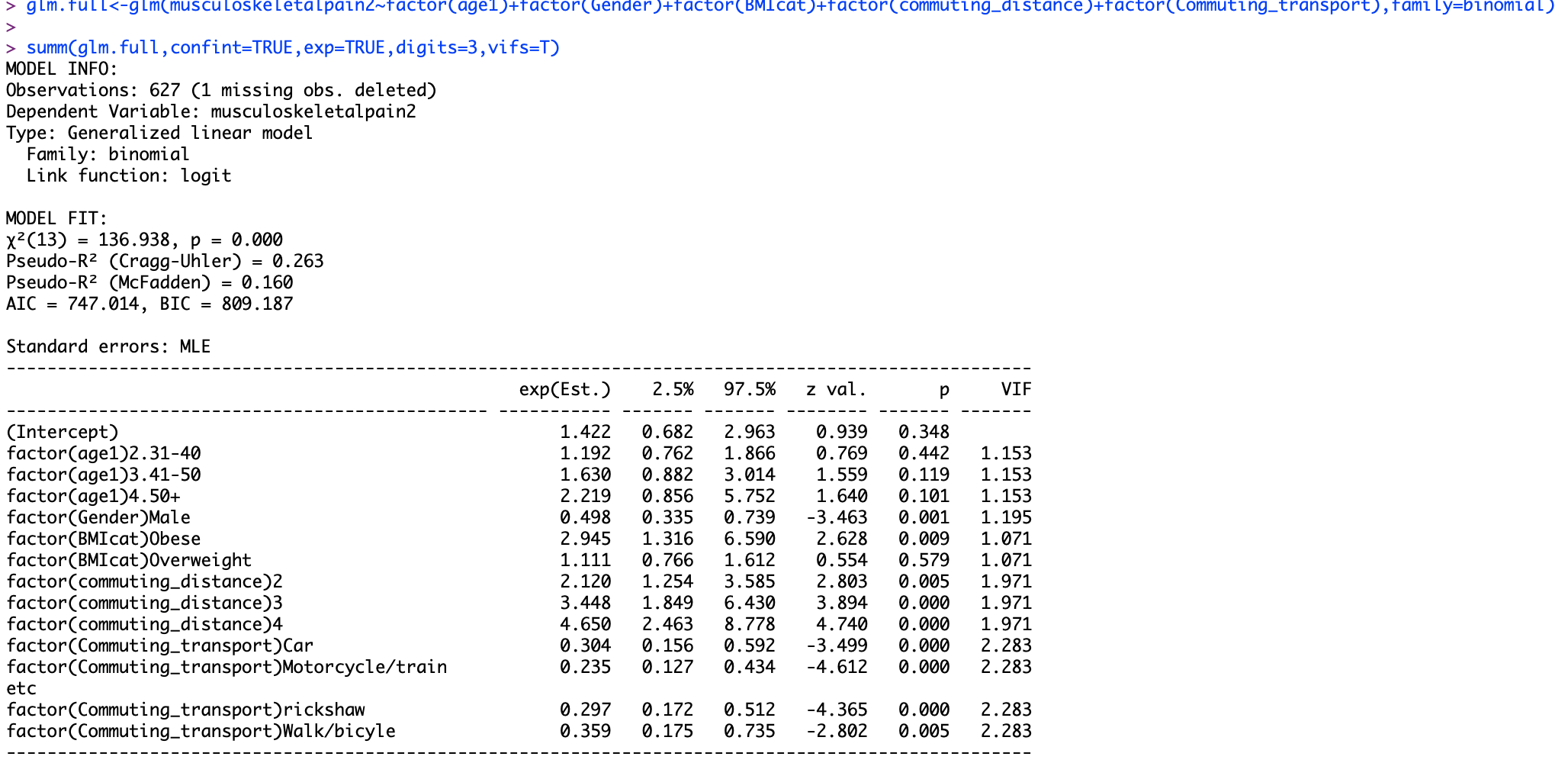


Note: Commuting distance 2=2-5 km 3=5-8 km and 4= above 8 km

# S5: Multivariable logistic regression model: Adjustment of confounders including traffic congestion experience to workplace


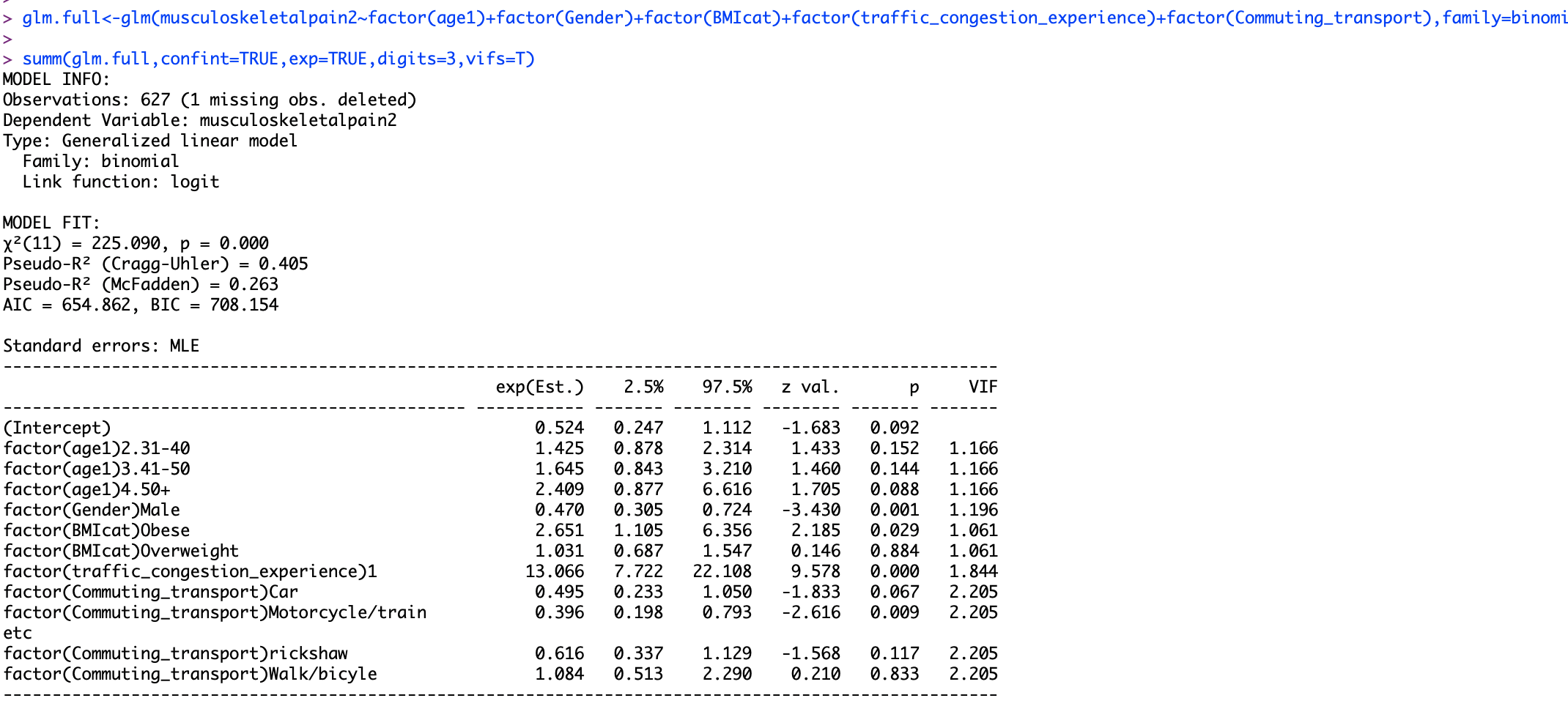

Supplement: Supplementary file 1 — Supplementary Material [file JOH2-63-e12289-s001.docx]
